# The use of immune checkpoint inhibitors in advanced gastric/gastroesophageal adenocarcinomas – real-world evidence and the use of alternative dosing

**DOI:** 10.3332/ecancer.2024.1741

**Published:** 2024-08-20

**Authors:** Aditya Dhanawat, Mehak Trikha, Manan Vora, Himanshu Gujarathi, Vikas Ostwal, Prabhat Bhargava, Rajiv Kaushal, Anant Ramaswamy

**Affiliations:** Department of Medical Oncology, Tata Memorial Hospital, Homi Bhabha National Institute (HBNI), Dr E Borges Road, Parel, Mumbai 400 012, India

**Keywords:** immunotherapy, checkpoint inhibitors, gastric cancer, low dose immunotherapy, real-world

## Abstract

**Background:**

Immune check point inhibitors (ICIs) have an established role in Microsatellite-Instability–High (MSI-H) and Combined Positive Score (CPS) high advanced gastric/gastroesophageal (G/GE) adenocarcinomas, but there is limited real world data with regard to practice patterns, and efficacy of standard doses (SD-ICIs) and alternative lower doses (LD-ICIs).

**Methods:**

A retrospective study of patients with advanced G/GE adenocarcinomas receiving ICIs was conducted. The primary endpoint of the study was 12-month overall survival (OS), which was computed by Kaplan-Meier method.

**Results:**

A total of 91 patients were available for analysis during the study period. Seventy-four patients (81%) received nivolumab, while the remaining received pembrolizumab. Fifteen patients (16%) had MSI-H status and had a 12-month OS of 60% and median OS of 15 months (median follow-up – 38.3 months). In the Microsatellite-Stable (MSS) cohort (84%; *n *= 76), ICIs (combined with chemotherapy) were used predominantly in pre-treated patients (54%; *n *= 41). Patients with CPS ≥5 (72%; *n *= 55) had improved survival compared to patients with CPS <5 (28%; *n *= 21) (12-month OS: 52% *vs*. 26%; Median OS: 12.8 months *vs*. 3.2 months; *p *= 0.005). There was no difference in survival between patients who received SD-ICIs (54%; *n *= 41) and LD-ICIs (46%; *n *= 35) (12-month OS: 42% *vs*. 48%; Median OS: 8.7 months *vs*. 11 months; *p *= 0.44).

**Conclusions:**

Patients with advanced G/GEJ adenocarcinomas in the real world predominantly received ICIs during later lines of therapy as opposed to first line therapy. Using a CPS cutoff of ≥5 as opposed to CPS <5 predicts for improved survivals in MSS patients and patients receiving low dose ICIs have similar survival outcomes to patients receiving standard dose ICIs within the confines of a heterogenous study cohort.

## Introduction

Patients with advanced gastric/gastroesophageal (G/GE) adenocarcinoma have been treated with 5-FU/capecitabine and platinum (cisplatin or oxaliplatin) based regimens as the backbone of therapy [1, 2]. Targeted agents against HER2 and claudlin 18.2 have additionally shown efficacy when combined with chemotherapy, thereby improving overall survival (OS) [3, 4].

The efficacy of pembrolizumab in microsatellite instability high (MSI-H)/mismatch repair deficient advanced G/GEJ adenocarcinomas has been established by the results of the phase 2 KEYNOTE-158 study (comprising 10.3% of patients with G/GEJ adenocarcinomas) [5]. Additionally, nivolumab has shown efficacy in combination with chemotherapy compared to chemotherapy alone in untreated G/GEJ adenocarcinomas with the majority of the survival improvements noted as the combined positive score (CPS) increases [6, 7].

While immune checkpoint inhibitors (ICIs) have been shown to improve survivals in advanced G/GEJ adenocarcinomas, their use in the real world, especially in lower middle-income countries (LMICs), is fraught with financial implications due to the relatively expensive nature of ICIs. This may result in patients in LMICS receiving ICIs outside standard trial criteria (as second-line therapy or in combination with other chemotherapy backbones besides FOLFOX/CAPOX/SOX) or at lower than approved doses. The use of ICIs in such scenarios will unlikely be explored in randomised clinical trials and hence, practice patterns and outcomes from real-world practice can provide rational for or against the use of ICIs on a larger scale. With this background, we retrospectively evaluated patients who would fit into standard trial criteria and did not satisfy trial criteria with regard to the usage of ICIs in patients with advanced G/GEJ adenocarcinomas.

## Materials and methods

### Patient selection

The current retrospective study aimed to evaluate the survivals of patients with advanced G/GEJ adenocarcinomas who were treated with ICIs, either as monotherapy or in combination with chemotherapy. The investigators evaluated data from a prospectively maintained gastric cancer database at Tata Memorial Hospital (TMH) and included patients who had been treated between January 2017 and January 2022. Patients included in the study satisfied the following criteria: histologically confirmed G/GE adenocarcinoma; radiologically confirmed unresectable or metastatic cancer; received at least one dose of ICIs as first-line therapy or during later lines of therapy; had at least one follow-up visit documenting response post administration of the ICIs, and had documented dates of starting and cessation of ICIs.

### Clinical data collection

Data collected were demographic and clinical variables, disease-specific data including the HER2 status, PD-L1 status, CPS, MMR (or MSI), details of ICIs administered, adverse events and oncologic outcomes. As per institutional protocol, HER2, PD-L1, CPS and MMR status was determined at baseline prior to initiating systemic therapy. The primary endpoint of the study was 12-month OS, while secondary endpoints were median OS, 6-month OS, progression-free survival (PFS), 6-month PFS, overall response rates (ORR) and adverse event rates.

### Ethics and consent

The approval for the study was obtained from the Institutional Ethics Committee at TMH; IEC418. The approval included the requirement of a short telephonic consent for patient data accrued in TMH as part of ethics committee requirements. Data collection and handling were conducted as per the ethical guidelines of the Declaration of Helsinki.

### Statistics

Data were analysed using IBM SPSS version 20 (Armonk, NY). Descriptive statistics such as median, frequency and percentage were used to summarize the categorical variables. Patients in the study were divided into three cohorts – cohort 1 (MSI -H), which included patients receiving ICIs, irrespective of ICIs as initial therapy or during later lines of therapy; cohort 2 (trial like), which included treatment-naïve Microsatellite Stable (MSS) patients receiving a standard dose of ICIs (nivolumab or pembrolizumab) in combination with FOLFOX/CAPOX/FLOT as first-line therapy with an ECOG PS of 0 or 1, and cohort 3 (Real-world), which included predominantly included patients who did not satisfy at least one of the above criteria, i.e. had received prior systemic therapy, received low-dose ICIs (LD-ICI) or administered a chemotherapy backbone besides FOLFOX, CAPOX or FLOT or had ECOG PS ≥2. For the purposes of the study, standard doses were considered as follows – Pembrolizumab 200 mg every 3 weeks, Nivolumab 3 mg/kg every 2 weeks (in MSI-H cancers), Nivolumab 240 mg every 2 weeks or 360 mg every 3 weeks when concurrently administered with chemotherapy. Doses lower than those previously mentioned were labeled as LD-ICIs and included dosing schedules such as Nivolumab at 20 and 40 mg every 2 or 3 weeks. The primary end point of the study was 12-month OS, which was the proportion of patients alive at 12 months, calculated from the date of starting ICIs (with or without chemotherapy) as measured by Kaplan-Meier estimate. Secondary endpoints were median OS, which was calculated from the date of starting ICIs (with or without chemotherapy) to the date of death or loss of follow-up, whichever was earlier; PFS, which was calculated from the date of diagnosis of starting ICIs to the date of progression, loss to follow-up or death, whichever was earlier; 6-month OS, which was the proportion of patients alive at 6 months, calculated from the date of starting ICIs (with or without chemotherapy) as measured by Kaplan-Meier estimate; 6-month PFS, which was the proportion of patients without disease progression 6 months, calculated from the date of starting ICIs (with or without chemotherapy) as measured by Kaplan-Meier estimate; and ORR, which were calculated by combining complete response (CR) and partial response (PR) rates, while clinical benefit rate was reported as a summation of CR, PR and stable disease (SD) rates. Grades 3 and 4 toxicities as well as toxicities of special interest (immune-related adverse events (IRAE)) were recovered from medical records and reported as per National Cancer Institute-common terminology criteria for adverse events version 5.0. Survival analysis was performed using Kaplan-Meier estimates, and the log-rank test was used for bivariate comparisons. Prognostic factors with a *p* value of ≤0.05 on univariate analysis were considered as significant and evaluated for multivariate analysis.

## Results

### Baseline characteristics

A total of 91 patients received ICIs in the study period; 15 patients (16%) in cohort 1, 13 patients (14%) in cohort 2 and 63 patients (69%) in cohort 3. Briefly, in cohort 1, 80% of patients received ICIs after prior therapy and 53% of patients received standard full dose ICIs. In cohort 2, 78% of patients had a CPS ≥5 and the most common chemotherapy backbone was CAPOX (39%). In cohort 3, 71% of patients had a CPS ≥5, 65% of patients had received prior systemic therapy, 70% received a chemotherapy backbone besides FOLFOX, CAPOX or FLOT and 56% of patients received LD-ICIs. Detailed characteristics are mentioned in Table 1.

### Immune-related adverse events

IRAEs were noted in seven patients (11%) patients in the entire cohort, with the most common IRAE’s being hypothyroidism (11%) (Table 2).

### Response rates and survival

For patients in cohort 1, ORR was 33.3%, with 3 (20%) having CR and 2 had (13.3%) PR, respectively. In cohort 2, ORR was 69.2%, with zero patients having a CR and 9 (69.2%) PR while in cohort 3, ORR was 27% with 1 (1.6%) having CR and 16 (25.4%) had a PR (Supplementary Figure 1).

With a median follow up of 38.3 months (95% CI: 4.9–71.7), patients in cohort 1 (MSI-H) had a 12-month OS, median OS and 12-month PFS of 60%, 15 months and 60%, respectively. With a median follow up of 4.8 months (95% CI: 4.0–5.6), patients in cohort 2 (trial-like) had an estimated 6-month OS of 66.7% median OS not reached and a 6-month PFS of 33.4%. With a median follow up of 11.1 months (95% CI: 6–16.3), patients in cohort 3 (Real world) had a 12-month OS of 41.4%, a median OS of 9.3 months and an estimated 12-month PFS of 28.2%.

**Figure 1.**
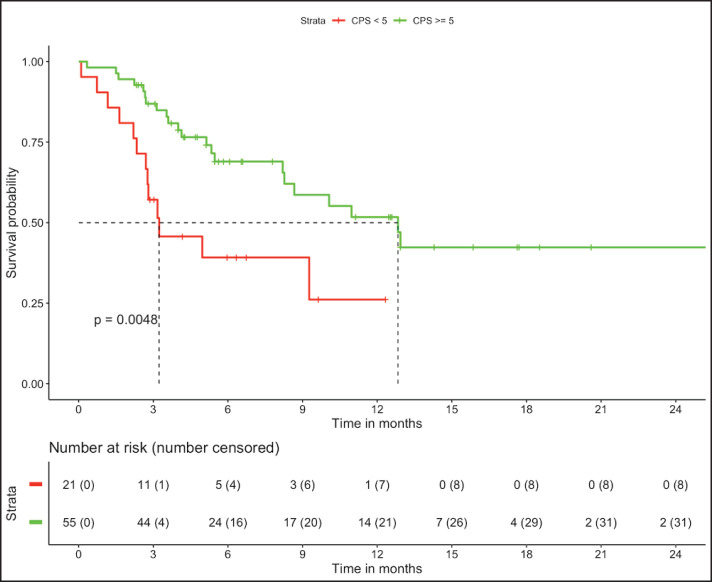
OS in non-MSI high patients on the basis of CPS.

**Figure 2.**
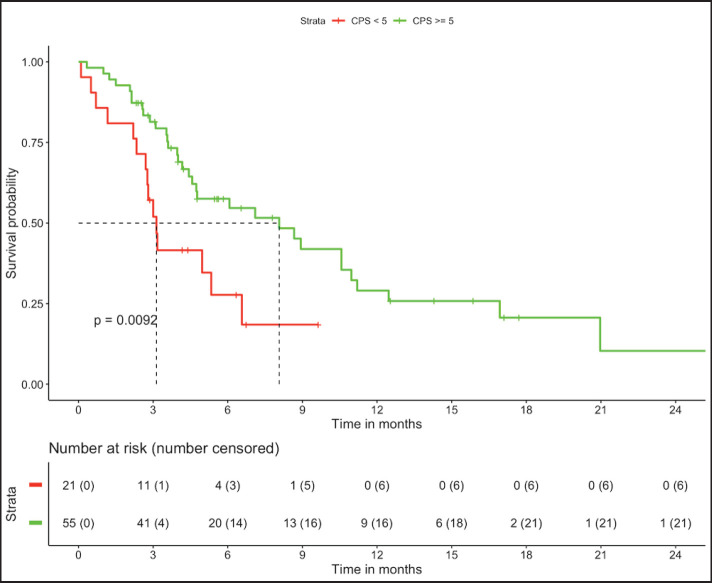
PFS in non-MSI high patients on the basis of CPS.

**Figure 3.**
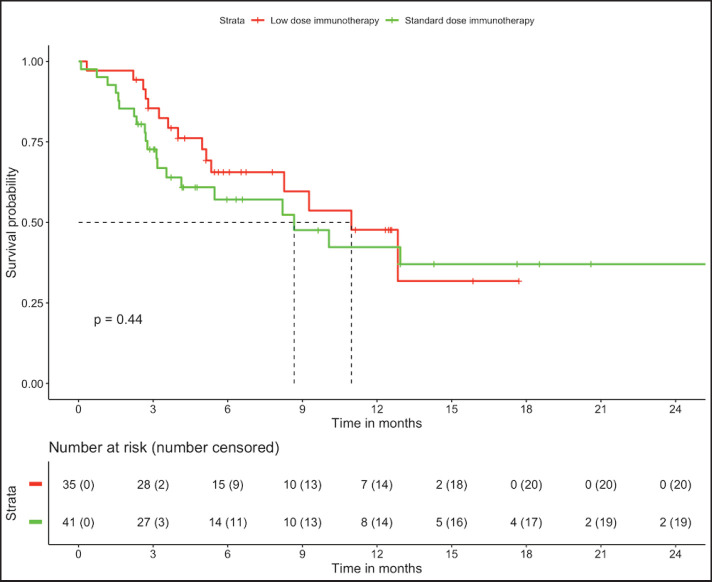
OS in non-MSI high patients on the basis of dose of immunotherapy.

**Figure 4.**
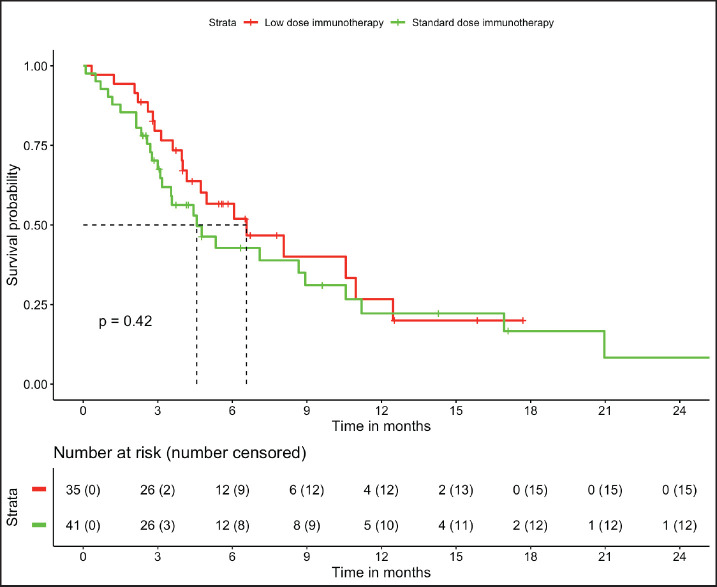
PFS in non-MSI high patients on the basis of dose of immunotherapy.

In patients with MSS (*n* = 76, trial-like and real world), patients with CPS ≥5 (*n* = 55) had significantly improved 6-month PFS (58% versus 28%, *p* = 0.009) and 12-month OS (52% versus 26%, *p* = 0.005) compared to patients with CPS <5 (*n* = 21). The corresponding median OS and median PFS for patients with CPS ≥5 was 12.8 (95% CI: 8.9–16.7) months and 8.1 (95% CI: 4–12.1) months, respectively, while the median OS and median PFS for patients with CPS <5 was 3.2 (95% CI: 0.6–5.9) months and 3.1 (95% CI: 2.6–3.6) months, respectively, (Figures 1 and 2). In patients with MSS, patients receiving LD-ICI (*n* = 35) had a 12-month OS of 48% and 12-month PFS of 27% while patients receiving SD-ICI (*n* = 41) had a 12-month OS and 12-month PFS of 42% and 22%. The corresponding median OS and median PFS for patients with LD-ICI was 11 (95% CI: 7.50–14.43) months and 6.6 (95% CI: 3–10.1) months, respectively, while the median OS and median PFS for patients with SD-ICI was 8.7 (95% CI: 3–14.3) months and 4.6 (95% CI: 2.4–6.7) months, respectively. There were no statistical differences in PFS (*p* = 0.42) or OS (*p* = 0.44) between patients receiving SD-ICIs and LD-ICIs (Figures 3 and 4).

## Discussion

The current study of patients with advanced G/GEJ adenocarcinomas receiving ICIs based on MSI status and CPS status is, to the best of authors’ knowledge, one of the earliest evaluating the use of ICIs in this scenario from an LMICS country. It throws light on the use of ICIs across scenarios, predominantly with regard to use beyond 1st line therapy, use of LD-ICI as well as usage in patients who do not strictly satisfy the inclusion and exclusion criteria used in seminal clinical trials.

The major reasons for dividing patients into three cohorts in the current study were three-fold. Primarily, patients with MSI-H cancers are exquisitely sensitive to ICIs and should not be bracketed with patients receiving ICIs for other indications such as high CPS or TPS scores [8, 9]. This was borne out by the results of the current study as well where patients with MSI cancers had improved survival (Median OS – 15 months) compared to other cohorts in the study. Second, the median follow-up in patients who received ICIs in combination with chemotherapy as first-line therapy formed a small proportion of patients in the study (14%) with a short median follow up (Less than 6 months). Finally, ICIs have shown relatively lesser efficacy in pre-treated patients, as seen in the KEYNOTE-061 study where pembrolizumab did not significantly improve survivals compared to paclitaxel [10]. Hence, the patients in the current study were separated into three cohorts and their outcomes not compared with each other.

Within the confines of such a heterogenous population, certain useful results can be gleaned. Even in patients who are predominantly pre-treated (65%) and ECOG PS 2 (25%) (real-world cohort), a median OS of approximately 9 months can be obtained and this is a reasonable outcome for this population. While nivolumab is currently approved in India for combination with chemotherapy irrespective of CPS scores, it is clear that the combination of ICIs and chemotherapy is predominantly beneficial when the CPS score is greater than or equal to 5. This was noted in the seminal clinical trials as well as the current study where patients with a CPS ≥5 had superior survivals compared to patients with CPS <5 [7, 11]. In an LMICS scenario, it is probably beneficial to consider the use of ICIs in patients who have a CPS of at least 5 as opposed to the blanket use of ICIs. However, it is prudent to mention that the use of ICIs in combination with chemotherapy in advanced gastric cancers is allowed in India irrespective of the CPS score. Besides efficacy, the overall incidence of IRAEs was low, though a proportion of MSS real-world patients (14%) receiving chemotherapy and ICIs did require dose modifications of chemotherapy due to grade 3 and 4 adverse events.

Another important finding is the fact that patients receiving LD-ICIs and SD-ICIs have similar survival outcomes when used in combination with chemotherapy in MSS patients. Though this is not an ideal comparison because of the heterogenous nature of the cohorts compared, it is important to note that MSS patients receiving LD-ICI were predominantly represented in the real-world cohort and this cohort had a significant proportion of patients with adverse prognostic factors such as ECOG PS 2, signet ring histology and being pre-treated. There is data emerging with regard to the efficacy of LD-ICIs and the results of this study are a valuable addition to the growing literature on this topic [12, 13]. Besides the clinical importance of these results, there are also major financial implications. The current patient support program for the use of nivolumab in patients with gastric cancer and MSS is approximately US$10700–US$14500 – this is not financially feasible in a majority of patients in India and is also not covered by government-sponsored schemes. The data from real-world studies such as ours cautiously provides some evidence that a greater number of patients can be brought be brought under the ambit of treatment with lower dose ICIs, though appropriately designed comparative clinical trials are needed before such practices are adopted in a widespread manner.

The major strength of our study lies in the real-world situation it reflects, with the important financial implications of a low-cost alternative to standard ICI dosing regimens besides providing reasonable survival outcomes. It also suggests that using a CPS cut-off of ≥5 might be a reasonable option in LMICS scenarios where financial considerations play a role. However, being a single institution retrospective study, there are a number of associated caveats that need to be kept in mind while analysing the results. The population studied is extremely heterogenous and the statistical comparisons made between these cohorts of patients is not ideal. MSS patients receiving ICIs as first-line therapy had an extremely short median follow up and we are unable to comment on any relevant long-term survivals in this cohort. We have analysed the data of patients receiving pembrolizumab and nivolumab together as a small proportion of patients receive ICIs in our clinical practice; however, both are distinct molecules. The use of LD-ICIs is primarily to overcome financial constraints in our patients – while preclinical studies do suggest similar activity between LD-ICIs and SD-ICIs, clinical trials comparing the same are clearly needed before this can be adapted to routine clinical practice.

In conclusion, patients with advanced G/GEJ adenocarcinomas in the real world predominantly received ICIs during later lines of therapy as opposed to first line therapy. Using a CPS cutoff of ≥5 as opposed to <5 seems to predict for improved survivals in MSS patients and patients receiving low dose ICIs have similar survival outcomes to patients receiving standard dose ICIs within the confines of a heterogenous study cohort. The results suggests that ICIs show reasonable survival outcomes in the real world and can be used in patients with alternative lower dosing than that used in seminal clinical trials.

## Conflicts of interest

None of the authors declare a conflict of interest.

## Funding

None.

## Author contributions

Concept and design – Dr Aditya Dhanawat, Dr Anant Ramaswamy, Dr Vikas Ostwal

Definition of intellectual content and literature search – All authors

Data acquisition, data analysis and statistical analysis – Dr Aditya Dhanawat, Dr Mehak Trikha, Dr Anant Ramaswamy, Dr Vikas Ostwal,

Manuscript preparation – Dr Aditya Dhanawat, Dr Anant Ramaswamy, Dr Vikas Ostwal

Manuscript editing and manuscript review – All authors

**Guarantor** – Dr Anant Ramaswamy

## Statement of intent

The manuscript has been read and approved by all the authors, that the requirements for authorship as stated earlier in this document have been met, and that each author believes that the manuscript represents honest work.

## Figures and Tables

**Table 1.** Baseline characteristics of entire population.

Characteristic	MSI – H (%)	Trial-like cohort (%)	Real-world cohort (%)
Number of patients	15	13	63
Median age (years) (Range)	65 (35–76)	60 (43–75)	55 (27–78)
Male gender	12 (80)	10 (77)	41 (65)
Signet ring histology	2 (13)	1 (8)	11 (18)
Microsatellite-instability–high	15 (100)	0	0
HER2 positive	1 (7)	4 (31)	9 (14)
CPS <5 ≥5	12 (80) 3 (20)	3 (23) 10 (78)	18 (29) 45 (71)
Prior lines of systemic therapy 0 1 2 > 2	3 (20) 5 (33) 5 (33) 2 (13)	13 (100) 0 (0) 0 (0) 0 (0)	22 (35) 25 (40) 10 (16) 6 (10)
Sites of metastases Liver Lung Peritoneal Non-regional nodes Osseous	6 (40%) 5 (33.3%) 11 (73.3%) 11 (73.3%) 1 (6.7%)	6 (46.2%) 2 (15.4%) 4 (30.8%) 8 (61.5%) 2 (15.4%)	27 (42.9%) 11 (17.5%) 33 (52.4%) 46 (73%) 8 (12.7%)
ECOG PS 0 1 ≥2	0 12 (80) 3 (20)	0 13 (100) 0	1 (2) 46 (73) 16 (25)
Chemotherapy backbone FOLFOX CAPOX FLOT Others None	1 (7) 0 0 1 (7) 13 (87)	2 (15) 8 (62) 3 (17) 0 0	6 (10) 2 (3) 4 (6) 44 (70) 7 (11)
ICI used Nivolumab Pembrolizumab	11 (73) 4 (27)	7 (54) 6 (46)	56 (89) 7 (11)
ICI dosing Standard dose Low dose	8 (53) 7 (47)	13 (100) 0	28 (44) 35 (56)

ICI – Immune checkpoint inhibitor

**Table 2.** Treatment related adverse events of entire population.

Characteristic	MSI-H cohort (*N* = 15)	Trial-like cohort (*N* = 13)	Real-world cohort (*N* = 63)
Chemotherapy related grade 3 and 4 events			
Neutropenia	0	1 (8)	6 (10)
Thrombocytopenia	1 (7)	2 (15)	0
Anaemia	0	0	1 (2)
Mucositis	0	0	1 (2)
Vomiting	1 (7)	0	1 (2)
Diarrhoea	1 (7)	0	3 (5)
Neuropathy	0	0	3 (5)
HFS	0	0	6 (10)
Chemotherapy dose modifications	4 (27)	0	9 (14)
IRAE (all grades)			
Hypothyroidism	3 (20)	1 (8)	3 (5)
Skin reactions	0	0	1 (2)
Hepatitis	0	1 (8)	1 (2)
Pneumonitis	0	0	3 (5)
Others	0	0	1 (7)^#^

# acute appendicitis

**Supplementary Figure 1.**
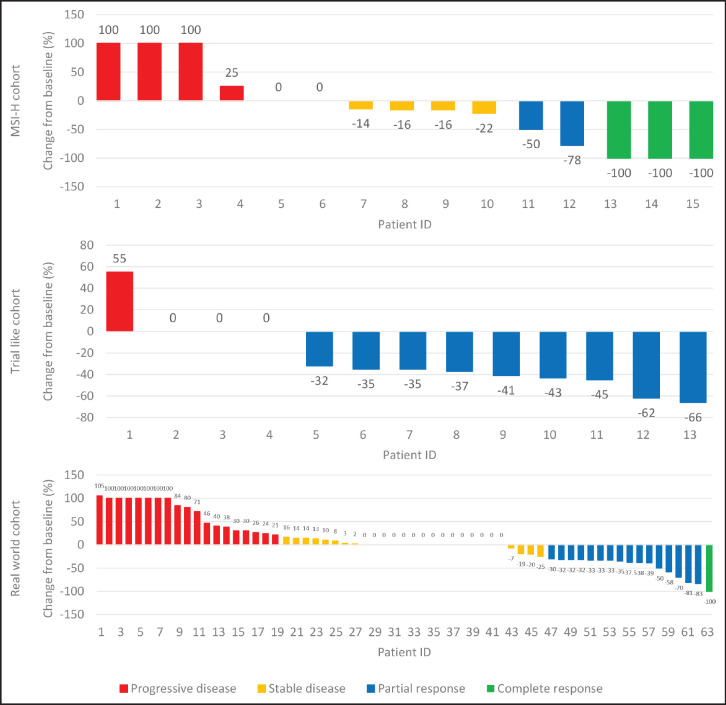
Waterfall plot: maximum tumor change from baseline by the best overall response in the MSI-H, Trial like cohort and Real world cohort.
